# Genetically Predicted Frailty Index Is Associated With Increased Risk of Multiple Metabolic Diseases: 175 226 European Participants in a Mendelian Randomization Study

**DOI:** 10.1111/1753-0407.70062

**Published:** 2025-03-02

**Authors:** Hexing Wang, Haifeng Zhang, Dongliang Tang, Yinshuang Yao, Junlan Qiu, Xiaochen Shu

**Affiliations:** ^1^ Department of Epidemiology, School of Public Health Suzhou Medical College of Soochow University Suzhou People's Republic of China; ^2^ Department of Nutrition and Food Hygiene, Hubei Key Laboratory of Food Nutrition and Safety, MOE Key Laboratory of Environment and Health, School of Public Health Tongji Medical College, Huazhong University of Science and Technology Wuhan People's Republic of China; ^3^ Department of Oncology and Hematology, Suzhou Hospital, Affiliated Hospital of Medical School Nanjing University Nanjing People's Republic of China; ^4^ Jiangsu Key Laboratory of Preventive and Translational Medicine for Major Chronic Non‐Communicable Diseases, MOE Key Laboratory of Geriatric Diseases and Immunology Suzhou Medical College of Soochow University Suzhou Jiangsu People's Republic of China

**Keywords:** frailty index, mendelian randomization, metabolic diseases

## Abstract

**Background:**

A relationship between frailty index (FI) and metabolic diseases (MDs) has been reported in previous observational studies. However, the causality between them remains unclear. This study aimed to examine the causal effect of FI on MDs.

**Methods:**

We performed a bidirectional two‐sample Mendelian randomization (MR) study. A recent large‐scale genome‐wide association study (GWAS) provided available data associated with FI, and summary statistics on eight MDs were collected from the IEU OpenGWAS database. Inverse variance weighted (IVW) was used as the main analysis to estimate causal effects, together with MR pleiotropy residual sum and outlier (MR‐PRESSO), MR‐Egger, Cochran's Q test, pleiotropy test, leave‐one‐out method, and MR Steiger analysis were used in the sensitivity analyses.

**Results:**

Our MR study demonstrated for the first time that elevated FI was causally associated with an increased risk of MDs including obesity (odds ratio [OR] = 1.78; 95% confidence interval [CI]: 1.17–2.70; *p* = 0.0075), T2DM (OR = 1.67; 95% CI: 1.24–2.24; *p* = 6.95 × 10^−4^), gout (OR = 2.45; 95% CI: 1.29–4.64; *p* = 0.006), hypothyroidism (OR = 1.96; 95% CI: 1.47–2.60; *p* = 3.47 × 10^−6^), and HTN (OR = 2.17; 95% CI: 1.72–2.74; *p* = 5.25 × 10^−11^). However, no causal association was found between FI and osteoporosis, vitamin D deficiency, and hyperthyroidism.

**Conclusions:**

Our findings support a causal relationship between FI and multiple MDs. This is crucial for the prevention of associated MDs in patients with frailty.

**Abbreviations**
CI: confidence interval
FI: frailty index
GWAS: genome‐wide association studies
HTN: hypertension
IEU: Integrative Epidemiology Unit
IV: instrumental variable
IVW: inverse‐variance weighted
LD: linkage disequilibrium
MDs: metabolic diseases
MR: Mendelian randomization
MR‐PRESSO: MR pleiotropy residual sum and outlier
OR: odds ratio
OS: osteoporosis
RCT: Randomized controlled trials
SNP: single nucleotide polymorphism
T2DM: type 2 diabetes mellitus
VDD: vitamin D deficiency
WMM: Weighted median method

**Summary**

- Aim: Using Mendelian randomization to explore causal associations between the frailty index and multiple metabolic diseases from a genetic perspective.
- Findings: Increased frailty index was causally associated with an increased risk of obesity, type 2 diabetes, gout, hypothyroidism, and hypertension.
- Message: Frailty may trigger an increased risk of developing a variety of diseases, and the early interventions implemented can help patients avoid more serious diseases.

## 1 Introduction

Frailty is closely associated with aging and is characterized by increased susceptibility to sudden, disproportionate decline in function following stressful events, often associated with a decline in the physiological capacity of multiple organ systems, which increases the likelihood of an individual's dependence on care or death [1]. Frailty is commonly measured by the Frailty Index (FI), which defines frailty as the cumulative effect of an individual's impairments and includes 92 baseline parameters for symptoms (e.g., depressed mood), signs (e.g., tremor), laboratory outliers, disease states, and disabilities (collectively referred to as impairments) [2]. The FI has been validated in several studies and has been shown to be more appropriate than other measures for assessing frailty [3].

Metabolic diseases (MDs) are a group of disorders in which the body's substance metabolism or energy metabolism is abnormal and exhibits metabolic disorders, usually systemic multi‐system abnormalities. The incidence of MDs has been increasing over the past few decades, placing a heavy burden and negative impact on both the health and finances of patients [4]. Eight metabolic diseases, including obesity [5], type 2 diabetes mellitus (T2DM) [6], osteoporosis (OS) [7], vitamin D deficiency (VDD) [8], gout [9], hyperthyroidism [10], hypothyroidism [11], and hypertension (HTN) [5] were selected for analysis in our study.

A few epidemiological studies have linked frailty with MDs such as obesity [12] and T2DM [13], OS [14]. However, the exact causal relationship between the two is unclear. It is often claimed that randomized controlled trials (RCTs) are the most reliable method to establish causality [15]. However, conducting these trials may not always be a viable option due to cost, impracticality, or ethical issues. Mendelian randomization analysis is a viable research strategy for assessing potential causal relationships between exposures and outcomes in epidemiological studies [16, 17]. In MR, the use of genetic variants that are closely associated with exposure to estimate causal effects on outcomes can better address the aforementioned limitations of observational studies [18, 19].

We conducted the first comprehensive bidirectional two‐sample Mendelian randomization study assessing potential causal associations between frailty and the risk of eight MDs in the European population.

## 2 Methods

### 2.1 Study Design

**FIGURE 1:**
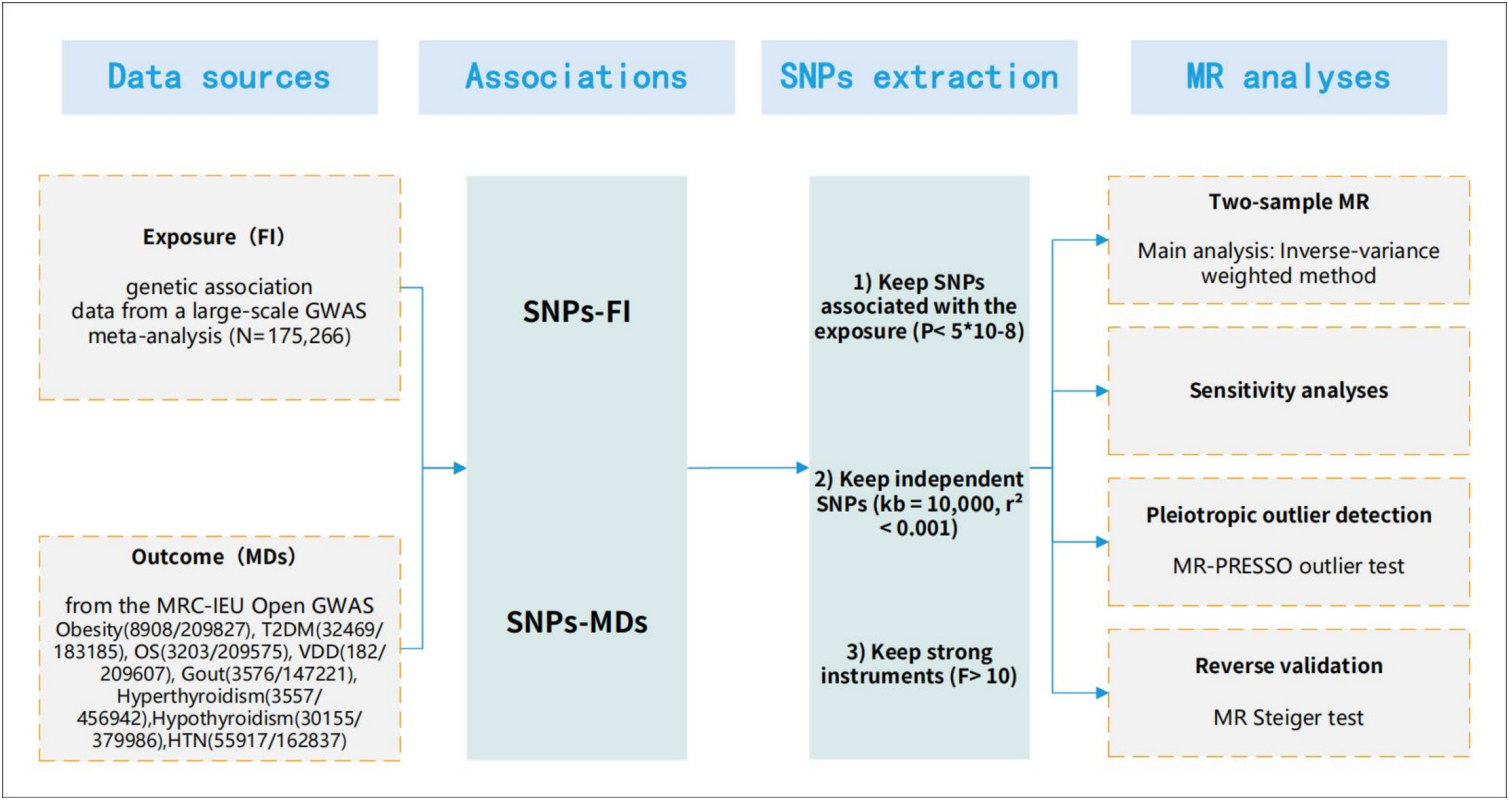
Design of the two‐sample MR study of the association between frailty index and metabolic diseases. Abbreviation: FI, frailty index; GWAS, genome‐wide association study; HTN, hypertension; MDs, metabolic diseases; MR, mendelian randomization; MR‐PRESSO, Mendelian Randomization Pleiotropy Residual Sum and Outlier; OS, osteoporosis; SNP, single nucleotide polymorphism; T2DM, type 2 diabetes mellitus; VDD, vitamin D deficiency.

The purpose of this MR study was to investigate the causal relationship between FI and MDs from a genetic standpoint. Figure 1 showed the design of our two‐sample MR study. To ensure that causal estimates from MR studies are valid, three core instrumental variable (IV) assumptions should be met (Figure S1) [20]: (1) genetic variation should be associated with exposure; (2) genetic variation should be independent of confounders associated with the exposure–outcome association; and (3) genetic variation should not affect outcomes independently of exposure.

### 2.2 Ethics Statement

All studies included in the cited GWAS have been approved by the relevant review committees. All participants had provided informed consent.

### 2.3 GWAS Data Source for the Frailty Index

Fourteen genetic variants significantly associated with FI were provided by a large GWAS meta‐analysis (Table 1) that included 175 226 European participants from the UK Biobank (*n* = 164 610, aged 60–70 years) and TwinGene (*n* = 10 616, aged 41–87) [21].

**TABLE 1:** Genetic instruments for the frailty index.

SNP	CHR	Effect_allele	Other_allele	EAF	Beta	se	F‐statistics	*p*.
rs10891490	11	C	T	0.5915	−0.0188	0.0034	31	2.00E‐08
rs12739243	1	C	T	0.2206	−0.0242	0.004	37	1.28E‐09
rs1363103	5	C	T	0.38	−0.0191	0.0034	32	2.23E‐08
rs17612102	15	C	T	0.5933	0.0187	0.0034	30	2.85E‐08
rs2071207	3	C	T	0.478	−0.0187	0.0033	32	1.47E‐08
rs2396766	7	A	G	0.4725	0.0201	0.0033	37	1.22E‐09
rs3959554	15	G	A	0.4177	0.0189	0.0034	31	1.74E‐08
rs4146140	10	T	C	0.3811	−0.0198	0.0034	34	6.83E‐09
rs4952693	2	T	C	0.3734	−0.0194	0.0034	33	1.47E‐08
rs56299474	8	A	C	0.1733	0.0241	0.0044	30	3.94E‐08
rs583514	3	C	T	0.5111	0.0199	0.0033	36	1.65E‐09
rs8089807	18	T	C	0.1866	−0.0248	0.0043	33	6.50E‐09
rs82334	4	C	A	0.3177	−0.0223	0.0035	41	3.13E‐10
rs9275160	6	A	G	0.3397	0.0382	0.0035	119	7.18E‐28

Abbreviations: CHR, chromosome; EAF, effect allele frequency; se, standard error; SNP, single nucleotide polymorphism.

### 2.4 GWAS Data Sources for Eight Metabolic Diseases

To collect the most extensive and up‐to‐date information on outcomes in European populations, we selected the summary statistics for the eight MDs: obesity (8908/209827), T2DM (32 469/183185), OS (3203/209575), VDD (182/209607), gout (3576/147221), hyperthyroidism (3557/456942), hypothyroidism (30 155/379986), HTN (55 917/162837) were extracted from the MRC‐IEU Open GWAS data infrastructure (IEU OpenGWAS project (mrcieu.ac.uk)) (Table S1).

### 2.5 Selection of the Genetic Instruments

The confidence of MR causal effect estimates depends heavily on valid instrumental variables. We systematically selected genetic instruments following strict criteria: (1) First, 14 single nucleotide polymorphisms (SNPs) were significantly correlated with FI genome‐wide (*p* < 5 × 10^−8^) [22]. (2) To ensure the independence of SNPs as genetic instruments, we performed linkage disequilibrium (LD) clustering using PLINK software. A stringent cut‐off of *r* ^2^ < 0.001 and 10 000 kb was applied using the European genotype data from the 1000 Genomes Project as a reference panel [23, 24]. (3) The effects of SNPs on exposure and outcome were reconciled to ensure that *β* values were signed to the same effector allele and then removed with an intermediate allele frequency (> 0.42) [25], (4) We used radial MR heterogeneity tests (modified Q‐statistics) [26] and MR‐PRESSO outlier tests [27], with a *p* value threshold of 0.05. If outliers appeared, the remaining SNPs after culling were used for the subsequent analyses.

### 2.6 Testing the Strength and Statistical Power of Genetic Instruments

The F‐statistic, derived from the formula: (*β*_exposure_ × *β*_exposure_)/ (se_exposure_ × se_exposure_) [28], was used to evaluate the strength of the FI genetic instruments. A cut‐off value of 10 was used to distinguish between strong and weak instruments, with a higher F‐statistic indicating a stronger instrument [29]. Statistical power in GWAS is determined by sample size, the proportion of cases in case–control GWAS, and the variation in exposure explained by genetic instruments.

### 2.7 Statistical Analyses and Sensitivity Analyses

In a preliminary MR analysis, we used an inverse variance weighted (IVW) MR [30] approach to estimate the association between FI and the risk of MDs in the European population. IVW is divided into two methods: random effects and fixed effects. This approach obtained the final causal effect estimates by weighting the combination of effect estimates for each instrumental variable. The weights were calculated based on the inverse of the variance of the effect estimates for each instrumental variable. This weighting made the estimates more accurate. MR‐Egger [31], Weighted median method (WMM) [32], and Maximum likelihood MR method [33] were used as a complementary methods for multiple validation. *p* values, odds ratios (OR), and their 95% confidence intervals (CI) were used to measure causal effects. A major advantage of the MR‐Egger method is its ability to initially determine the presence of horizontal multivariance by testing whether the intercept term is zero. The WMM is relatively robust to the presence of some null instrumental variables (IVs that do not meet the assumptions) because it is based on the concepts of ordinal and median, rather than directly weighting each estimate as in the IVW approach, which reduces the impact of outliers or null estimates on the general results. The maximum likelihood MR method takes full advantage of the information in the data to more accurately estimate causality by constructing a likelihood function. Cochran's Q test [26] was used to test for heterogeneity between the genetic instruments in the main analysis; the presence of heterogeneity was indicated by a *p* < value of 0.05. If there was no significant heterogeneity, a fixed‐effects IVW model was used; otherwise, a random‐effects IVW model was used.

As horizontal pleiotropy can bias effect estimates in MR, we performed MR‐Egger regression to assess potential pleiotropy via its intercept term. In addition, we specifically conducted the MR‐PRESSO global test, which assessed the overall level of pleiotropy between all IVs in a single MR test by comparing the observed distances of all variants from the regression line (sum of squares of residuals) to the expected distance under the null hypothesis of no level of pleiotropy. We also used pleiotropy_test to aid in verifying the presence of pleiotropy; *p* < 0.05 indicated significant evidence of potential directed pleiotropy. The leave‐one‐out methods [34] observed whether the results changed after removing each SNP by gradually removing each SNP and calculating the meta‐effect of the remaining SNPs. If the results demonstrated minimal variation, this suggested that they were robust. Scatterplots and forest plots were used as visualization treatments.

Furthermore, we comprehensively screened the 14 SNPs that were used by utilizing the LDtrait [35] tool to assess associations with any previous potentially confounding features (psychological factors [36] [e.g., depression, anxiety, and chronic stress, etc.] and behavioral factors [37] [including quality of sleep, sedentary behaviors, smoking, and drinking, etc.]). MR analysis was repeated after removing these invalid SNPs to rule out possible residual pleiotropic effects. Finally, we further performed the MR Steiger test to investigate the potential reverse causal impact of FI on MDs [38].

To account for the issue of multiple testing, the *p* values were adjusted according to the method of Benjamini and Hochberg (B/H) [39] to control the false discovery rate (FDR). An association was deemed statistically significant if its corresponding Benjamini–Hochberg (B/H)‐adjusted *p* value was less than 0.05, corresponding to an FDR of 5%. An association with a nominal *p* value less than 0.05 and a B/H‐adjusted *p* value of 0.05 was considered a suggestive association. All *p* values were two‐sided. All statistical analyses and data visualization were performed in R software (version 4.1.3; R Development Core Team) with the packages TwoSampleMR, MR‐PRESSO, and forest plotter.

## 3 Results

### 3.1 Strength of Genetic Instruments

The F‐statistic calculated by the above formula must be greater than 10 to be used in subsequent analyses. Among these instrumental variables, the minimum F‐statistic was 30, indicating that all IVs were sufficient for preliminary MR analysis (Table 1).

### 3.2 Two‐Sample MR Analysis

**FIGURE 2:**
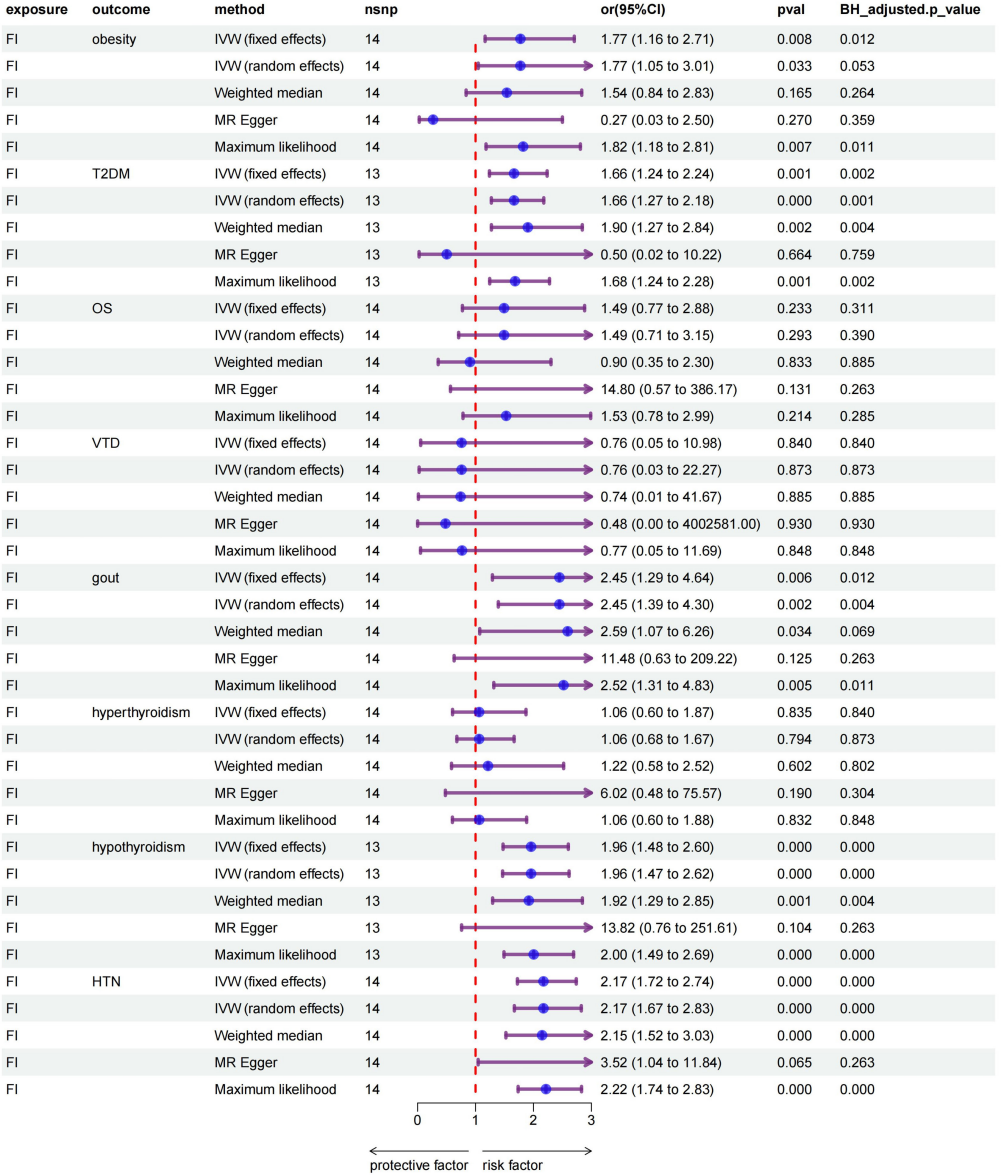
Forest plots displaying the identified causal effects of FI on MDs using five MR methods. Odds ratios, 95% CIs, pval, and BH_adjusted. *p* value are reported for the causal effect of FI on obesity, T2DM, gout, hypothyroidism, and HTN.Abbreviation: CI, confidence interval; FI, frailty index; HTN, hypertension; IVW, inverse‐variance weighted; Nsnp, number of single‐nucleotide polymorphism; OR, odds ratio; pval, *p*‐value; T2DM, type 2 diabetes mellitus. All statistical tests were two‐sided. pval and BH_adjusted. *p* value < 0.05 were considered statistically significant. OR > 1 is a risk factor, and 0 < OR < 1 is a protective factor.

Based on fixed‐effects IVW estimates, genetically predicted elevated FI was causally associated with increased risk of obesity (OR, 1.78; 95% CI: 1.17–2.70; *p* = 0.0075), gout (OR, 2.45; 95% CI: 1.29–4.64; *p* = 0.006), and HTN (OR, 2.17; 95% CI: 1.72–2.74; *p* = 5.25 × 10^−11^). However, no significant causal relationship was found between FI and OS (OR, 1.49; 95% CI: 0.77–2.88; *p* = 0.233), VDD (OR, 0.76; 95% CI: 0.05–10.98; *p* = 0.840) and hyperthyroidism (OR, 1.06; 95% CI: 0.60–1.87; *p* = 0.835). In addition, results remained valid after the Benjamini–Hochberg adjustment. Comprehensive information regarding the results of the MR analyses is available in the Table S2. A forest plot summarizing the main results is shown in Figure 2.

### 3.3 Validation and Sensitivity Analyses

In the first analysis, although all five MR analyses showed a significant causal effect of FI on T2DM and hypothyroidism, the results of the sensitivity analyses suggested horizontal pleiotropy. Therefore, we used the MR‐PRESSO difference‐in‐value test; the genetic locus rs9275160 was found to have a large effect on the results. After its exclusion, the previous analysis was repeated for the remaining 13 SNPs, at which point frailty had a causal effect on T2DM (OR, 1.67; 95% CI: 1.24–2.24; *p* = 6.95 × 10^−4^), hypothyroidism (OR, 1.96; 95% CI: 1.47–2.60; *p* = 3.47 × 10^−6^), and eliminated the effect of horizontal pleiotropy. Beyond that, no horizontal pleiotropy was detected in the present MR analysis (MR‐PRESSO global test: *p* > 0.05; The results of the sensitivity analysis were shown in Table 2). Moreover, the leave‐one‐out analysis indicated that no single instrument can dominate the pooled causal effect (Figure S3).

**TABLE 2:** Tests for polytropy and heterogeneity.

Exposure	Outcome	MR‐PRESSO	Cochran‐Q test	Q_pval	Pleiotropy_test
FI	Obesity	0.097	20.43977	0.08477419	0.1144663
FI	T2DM	0.63	9.963202	0.6191891	0.4504961
FI	gout	0.717	10.09875	0.6858401	0.3056639
FI	Hypothyroidism	0.459	12.43196	0.4116411	0.2117337
FI	HTN	0.273	16.76738	0.2101577	0.4405901

*Note:* MR‐PRESSO, Q_pval, and pleiotropy_test results > 0.05 indicated the absence of heterogeneity and horizontal pleiotropy.

Abbreviations: FI, frailty index; HTN, hypertension; MR‐PRESSO, Mendelian Randomization of Multi‐Randomized Residuals and Outliers; Q_pval, Q_*p* value; T2DM, type 2 diabetes mellitus.

Further conservative MR analysis was implemented to prevent the influence of residual pleiotropy by removing SNPs associated with potentially confounding traits (Table S4) and still got a similar estimate for obesity (OR, 2.09; 95% CI: 1.31–3.35; *p* = 0.002), T2DM (OR, 1.52; 95% CI: 1.09–2.14; *p* = 0.014), gout (OR, 2.74; 95% CI: 1.35–5.59; *p* = 0.005), hypothyroidism (OR, 1.97; 95% CI: 1.42–2.72; *p* = 0.000) and HTN (OR, 2.17; 95% CI: 1.68–2.81; *p* = 0.000) (Table S5). Finally, we used the MR Steiger test to determine the direction of the causal effect, and the results confirm that the instruments of FI affected the susceptibility of MDs rather than the reverse and that the direction of the effect was robust (Table S3).

## 4 Discussion

To investigate the causal relationship between frailty and a multiple of metabolic diseases, we conducted this bidirectional, two‐sample MR study based on the European population and large‐scale GWAS summary statistics. In our analyses, the FI was used to assess the degree of frailty. The results of the MR analyses indicated significant causal associations between frailty and metabolic diseases, including obesity, T2DM, gout, hypothyroidism, and HTN. Validation and sensitivity analyses confirmed our findings, while no significant associations were found in the opposite direction.

Mechanistic issues between frailty and (MDs) need to be explored. Dr. Westbrook [40], Johns Hopkins University, USA, used liquid chromatography‐mass spectrometry to identify energy metabolism‐related products and pathways in the serum of a community cohort and explored their association with physiological frailty and risk of adverse outcomes. The results suggested that aging and frailty were accompanied by specific and measurable differences between glucose metabolism, the TCA cycle, and neurotransmitter metabolic intermediates [41]. This study contributed to further understanding of the targets of metabolic dysregulation in aging and physiological frailty. Andrew Clegg [42] found that the brain and the endocrine system were inextricably linked through the hypothalamic–pituitary axis, which controlled metabolism through the signaling of several homeostatic hormones, and played an important role in the regulation of aging and frailty.

Several previous epidemiological studies have shown an association between frailty and a multitude of metabolic diseases. For instance, in a prospective analysis that included 493 737 UK Biobank participants, frailty was assessed using the frailty phenotype and found a significant association between frailty and obesity [43]. Xingqi Cao et al. [44] conducted a prospective cohort study in which they concluded that frailty was positively associated with the risk of developing T2DM even at very early stages. A study on diabetes and frailty by Mariam El Assar et al. [45] showed that diabetes was highly prevalent in frail elderly patients, leading to further impairment of physical functioning in this population. The association between frailty and gout has been poorly studied, but several potential causes could explain the causal relationship. Patients with frailty often have difficulty maintaining a healthy lifestyle and lack effective physical activity, and Zhi Cao et al. [46] suggested that sedentary time was associated with a variety of adverse health conditions, including thyroid disease, depression, migraines, and gout. A study by Yuan Zhang et al. [47] also suggested that healthy lifestyles were associated with a lower risk of gout and may reduce the risk of gout associated with genetic factors by nearly one‐third. A cross‐sectional study of community‐dwelling older adults [48] demonstrated that subclinical hypothyroidism, elevated TSH levels, decreased fT3 levels, and decreased fT3/fT4 ratio were all associated with frailty in community‐dwelling older adults. A study by Teresa Gijón‐Conde et al. [49] showed that in the elderly, frailty was associated with cardiovascular risk factors, including HTN [50, 51].

The present study has several advantages. To our knowledge, this is the first bidirectional MR study assessing the association between frailty and the risk of multiple MDs using large‐scale genomic data from a European population. We selected genetic instrumental variables using strict criteria to minimize pleiotropy, performed a series of validation and sensitivity analyses accounting for null instruments, pleiotropy, and outliers to obtain reliable results.

Our study has several limitations. Most GWAS statistics were from individuals of European ancestry, so results may not generalize to other populations. Pleiotropy influenced our conclusions despite efforts to minimize it. Unrecognized pathways and confounders between exposure and outcome variables may bias our results.

In conclusion, this study provides sufficient evidence of a causal relationship between frailty and common metabolic diseases, including obesity, T2DM, gout, hypothyroidism, and HTN. It helps patients with frailty to seek individualized management and early intervention to avoid more serious diseases.

## Author Contributions

Conception and design: Hexing Wang, Haifeng Zhang, Dongliang Tang, Yinshuang Yao, and Xiaochen Shu. Financial support: Xiaochen Shu. Provision of study materials for patients: publicly available. Collection and assembly of data: Hexing Wang, Dongliang Tang, and Yinshuang Yao. Data analysis and interpretation: Hexing Wang, Haifeng Zhang, Dongliang Tang, Yinshuang Yao, and Junlan Qiu. Manuscript drafting: Hexing Wang, Haifeng Zhang, Dongliang Tang, Yinshuang Yao, Junlan Qiu, and Xiaochen Shu. Final approval of manuscript: Hexing Wang, Haifeng Zhang, Dongliang Tang, Yinshuang Yao, Junlan Qiu, and Xiaochen Shu.

## Ethics Statement

All studies included in cited genome‐wide association studies have been approved by the relevant review committees.

## Consent

All participants provided informed consent.

## Conflicts of Interest

The authors declare no conflicts of interest.

## Supporting information


**Data S1.** Supporting Information.

**Figure S1.** Core assumptions of Mendelian randomization.
**Figure S2.** Scatter plots of frailty index for obesity, type 2 diabetes, gout, hypothyroidism, and hypertension.
**Figure S3.** leave‐one‐out plots of frailty index for obesity, type 2 diabetes, gout, hypothyroidism, and hypertension.
**Table S1.** GWAS Data Sources for eight metabolic diseases.
**Table S2.** The results of Two‐sample MR analysis.
**Table S3.** The results of the MR Steiger test.
**Table S4.** Frailty‐related SNPs that associated with potential confounding traits identified in previous studies by LDtrait Tool.
**Table S5.** Conservative Mendelian randomization analysis by further removing SNPs associated with potential confounding traits.

## Data Availability

All summary genetic data utilized in the present study are available online from the corresponding genome‐wide association studies. All codes are available upon reasonable request by contacting the corresponding author (email: xcshu@suda.edu.cn).
