# Supplementary material for: Genetically Predicted Frailty Index Is Associated With Increased Risk of Multiple Metabolic Diseases: 175 226 European Participants in a Mendelian Randomization Study
Source: J Diabetes. 2025 Mar 2;17(3):e70062. doi: 10.1111/1753-0407.70062 (PMC11872387; doi:10.1111/1753-0407.70062)
Supplement: Supplementary file 2 — Figure S1. Core assumptions of Mendelian randomization. Figure S2. Scatter plots of frailty index for obesity, type 2 diabetes, gout, hypothyroidism, and hypertension. Figure S3. leave‐one‐out plots of frailty index for obesity, type 2 diabetes, gout, hypothyroidism, and hypertension. Table S1. GWAS Data Sources for eight metabolic diseases. Table S2. The results of Two‐sample MR analysis. Table S3. The results of the MR Steiger test. Table S4. Frailty‐related SNPs that associated with potential confounding traits identified in previous studies by LDtrait Tool. Table S5. Conservative Mendelian randomization analysis by further removing SNPs associated with potential confounding traits. [file JDB-17-e70062-s002.docx]

**Supplementary Files**

**Supplementary Table 1** GWAS Data Sources for eight metabolic diseases

**Supplementary Table 2**  The results of Two-sample MR analysis

**Supplementary Table 3**  The results of the MR Steiger test

**Supplementary Table 4**  Frailty-related SNPs that associated with potential confounding traits identified in previous studies by LDtrait Tool.

**Supplementary Table 5** Conservative Mendelian randomization analysis by further removing SNPs associated with potential confounding traits.

**Supplementary Figure 1** Core assumptions of Mendelian randomization.

**Supplementary Figure 2** Scatter plots of frailty index for obesity, type 2 diabetes, gout, hypothyroidism, and hypertension**.**

**Supplementary Figure 3** leave-one-out plots of frailty index for obesity, type 2 diabetes, gout, hypothyroidism, and hypertension**.**

| **Supplementary table 1 GWAS Data Sources for eight metabolic diseases** | | | | | | | |
| --- | --- | --- | --- | --- | --- | --- | --- |
| **Dataset** | **Year** | **Trait** | **Consortium** | **ncase** | **ncontrol** | **Number of** **SNPs** | **Population** |
| finn-b-E4_OBESITY | 2021 | obesity | NA | 8,908 | 209,827 | 16,380,465 | European |
| finn-b-E4_DM2 | 2021 | type 2 diabetes | NA | 32,469 | 183,185 | 16,380,440 | European |
| finn-b-M13_OSTEOPOROSIS | 2021 | osteoporosis | NA | 3,203 | 209,575 | 16,380,452 | European |
| finn-b-E4_VIT_D_DEF | 2021 | vitamin D deficiency | NA | 182 | 209,607 | 16,380,446 | European |
| finn-b-M13_GOUT | 2021 | gout | NA | 3,576 | 147,221 | 16,380,152 | European |
| ebi-a-GCST90018860 | 2021 | hyperthyroidism | NA | 3557 | 456,942 | 24,189,279 | European |
| ebi-a-GCST90018862 | 2021 | hypothyroidism | NA | 30,155 | 379,986 | 24,138,872 | European |
| finn-b-I9_HYPTENS | 2021 | Hypertension | NA | 55,917 | 162,837 | 16,380,466 | European |

Note: Abbreviations: GWAS, genome-wide association study; ncase, number of case; ncontrol, number of control; SNPs, single nucleotide polymorphisms.

| **Supplementary Table 2 The results of Two-sample MR analysis** | | | | | | | |  |
| --- | --- | --- | --- | --- | --- | --- | --- | --- |
| **exposure** | **outcome** | **method** | **nsnp** | ***p*** | **or** | **or_lci95** | **or_uci95** | **BH_adjusted p_value** |
| FI | obesity | IVW（fixed effects） | 14 | 0.008 | 1.78 | 1.17 | 2.70 | 0.012 |
| FI |  | IVW（random effects） | 14 | 0.033 | 1.78 | 1.05 | 3.01 | 0.053 |
| FI |  | Weighted median | 14 | 0.165 | 1.54 | 0.84 | 2.83 | 0.264 |
| FI |  | MR Egger | 14 | 0.270 | 0.27 | 0.03 | 2.50 | 0.359 |
| FI |  | Maximum likelihood | 14 | 0.007 | 1.82 | 1.18 | 2.81 | 0.0106 |
| FI | T2DM | IVW（fixed effects） | 13 | 0.001 | 1.67 | 1.24 | 2.24 | 0.002 |
| FI |  | IVW（random effects） | 13 | 0.000 | 1.67 | 1.27 | 2.18 | 0.001 |
| FI |  | Weighted median | 13 | 0.002 | 1.90 | 1.27 | 2.84 | 0.004 |
| FI |  | MR Egger | 13 | 0.664 | 0.50 | 0.02 | 10.22 | 0.759 |
| FI |  | Maximum likelihood | 13 | 0.001 | 1.68 | 1.24 | 2.28 | 0.002 |
| FI | OS | IVW（fixed effects） | 14 | 0.233 | 1.49 | 0.77 | 2.88 | 0.311 |
| FI |  | IVW（random effects） | 14 | 0.293 | 1.49 | 0.71 | 3.15 | 0.390 |
| FI |  | Weighted median | 14 | 0.833 | 0.90 | 0.36 | 2.30 | 0.885 |
| FI |  | MR Egger | 14 | 0.131 | 14.80 | 0.57 | 386.16 | 0.263 |
| FI |  | Maximum likelihood | 14 | 0.214 | 1.53 | 0.78 | 2.99 | 0.285 |
| FI | VDD | IVW（fixed effects） | 14 | 0.840 | 0.76 | 0.05 | 10.98 | 0.840 |
| FI |  | IVW（random effects） | 14 | 0.873 | 0.76 | 0.03 | 22.27 | 0.873 |
| FI |  | Weighted median | 14 | 0.885 | 0.74 | 0.01 | 41.67 | 0.885 |
| FI |  | MR Egger | 14 | 0.930 | 0.48 | 0.00 | 4002581.00 | 0.930 |
| FI |  | Maximum likelihood | 14 | 0.848 | 0.77 | 0.05 | 11.69 | 0.848 |
| FI | gout | IVW（fixed effects） | 14 | 0.006 | 2.45 | 1.29 | 4.64 | 0.012 |
| FI |  | IVW（random effects） | 14 | 0.002 | 2.45 | 1.39 | 4.30 | 0.004 |
| FI |  | Weighted median | 14 | 0.034 | 2.59 | 1.07 | 6.26 | 0.069 |
| FI |  | MR Egger | 14 | 0.125 | 11.48 | 0.63 | 209.22 | 0.263 |
| FI |  | Maximum likelihood | 14 | 0.005 | 2.52 | 1.32 | 4.83 | 0.011 |
| FI | hyperthyroidism | IVW（fixed effects） | 14 | 0.835 | 1.06 | 0.60 | 1.87 | 0.840 |
| FI |  | IVW（random effects） | 14 | 0.794 | 1.06 | 0.68 | 1.67 | 0.873 |
| FI |  | Weighted median | 14 | 0.602 | 1.21 | 0.59 | 2.52 | 0.802 |
| FI |  | MR Egger | 14 | 0.190 | 6.02 | 0.48 | 75.57 | 0.304 |
| FI |  | Maximum likelihood | 14 | 0.832 | 1.06 | 0.60 | 1.88 | 0.848 |
| FI | hypothyroidism | IVW（fixed effects） | 13 | 0.000 | 1.96 | 1.47 | 2.60 | 1.39E-05 |
| FI |  | IVW（random effects） | 13 | 0.000 | 1.96 | 1.47 | 2.62 | 2.05E-05 |
| FI |  | Weighted median | 13 | 0.001 | 1.92 | 1.30 | 2.85 | 0.004 |
| FI |  | MR Egger | 13 | 0.104 | 13.82 | 0.76 | 251.61 | 0.263 |
| FI |  | Maximum likelihood | 13 | 0.000 | 2.00 | 1.49 | 2.69 | 1.63E-05 |
| FI | HTN | IVW（fixed effects） | 14 | 0.000 | 2.17 | 1.72 | 2.74 | 4.20E-10 |
| FI |  | IVW（random effects） | 14 | 0.000 | 2.17 | 1.67 | 2.83 | 6.00E-08 |
| FI |  | Weighted median | 14 | 0.000 | 2.15 | 1.52 | 3.03 | 1.01E-04 |
| FI |  | MR Egger | 14 | 0.065 | 3.52 | 1.04 | 11.84 | 0.263 |
| FI |  | Maximum likelihood | 14 | 0.000 | 2.22 | 1.73 | 2.83 | 1.49E-09 |

Abbreviations: MR, Mendelian randomization; nsnp, number of single nucleotide polymorphisms; or, odds ratio; lci, lower confidence interval; uci, upper confidence interval; BH, Benjamini/Hochberg. FI, frailty index; IVW, inverse-variance weighted; T2DM, type 2 diabetes mellitus; OS, osteoporosis; VDD, vitamin D deficiency; HTN, hypertension.

| **Supplementary Table 3 The results of the MR Steiger test.** | | | | | |
| --- | --- | --- | --- | --- | --- |
| **Exposure** | **Outcome** | **R^2^ for exposure** | **R^2^ for outcome** | **Correct causal direction** | ***P*steige** |
| FI | Obesity | 0.003175157 | 0.000126253 | TRUE | 4.41E-45 |
| FI | T2DM | 0.003175157 | 0.000281972 | TRUE | 7.33E-35 |
| FI | Gout | 0.003175157 | 0.000116906 | TRUE | 1.58E-38 |
| FI | Hypothyroidism | 0.003175157 | 0.000635597 | TRUE | 8.35E-28 |
| FI | HTN | 0.003175157 | 0.000273317 | TRUE | 1.64E-35 |

Abbreviations: FI, frailty index; T2DM, type 2 diabetes mellitus; HTN, hypertension; R2, the variance of phenotype explained by genetic instruments.

**Supplementary Table 4**  **Frailty-related SNPs that associated with potential confounding traits identified in previous studies by LDtrait Tool.**

| **SNP** | **CHR** | **POS** | **Allele** | **Trait** |
| --- | --- | --- | --- | --- |
| rs12739243 | 1 | 210,302,043 | C/T | Smoking initiation (ever regular vs never regular) |
| rs3959554 | 15 | 41,443,924 | A/G | Coronary artery disease |
| rs1363103 | 5 | 103,917,837 | C/T | Insomnia |

**Abbreviations:** SNP, single nucleotide polymorphism; CHR, chromosome; POS, position.

| **Supplementary Table 5 Conservative Mendelian randomization analysis by further removing SNPs associated with potential confounding traits.** | | | | | | | |
| --- | --- | --- | --- | --- | --- | --- | --- |
| **Outcome** | **Method** | ***P-*value** | **OR** | **OR_lci95** | **OR_uci95** | ***P* for Heterogeneity** | ***P* for Pleiotropy** |
| Obesity | IVW（fixed effects） | 0.002 | 2.09 | 1.31 | 3.35 | 0.166 | 0.097 |
|  | IVW（random effects） | 0.009 | 2.09 | 1.20 | 3.66 |  |  |
|  | Weighted median | 0.097 | 1.76 | 0.90 | 3.45 |  |  |
|  | MR Egger | 0.295 | 0.30 | 0.04 | 2.49 |  |  |
|  | Maximum likelihood | 0.002 | 2.14 | 1.32 | 3.46 |  |  |
| T2DM | IVW（fixed effects） | 0.014 | 1.52 | 1.09 | 2.14 | 0.488 | 0.522 |
|  | IVW（random effects） | 0.012 | 1.52 | 1.10 | 2.12 |  |  |
|  | Weighted median | 0.025 | 1.70 | 1.07 | 2.69 |  |  |
|  | MR Egger | 0.669 | 0.43 | 0.01 | 17.71 |  |  |
|  | Maximum likelihood | 0.014 | 1.54 | 1.09 | 2.18 |  |  |
| OS | IVW（fixed effects） | 0.101 | 1.85 | 0.89 | 3.84 | 0.149 | 0.289 |
|  | IVW（random effects） | 0.174 | 1.85 | 0.76 | 4.46 |  |  |
|  | Weighted median | 0.354 | 1.61 | 0.59 | 4.43 |  |  |
|  | MR Egger | 0.189 | 14.35 | 0.37 | 563.87 |  |  |
|  | Maximum likelihood | 0.096 | 1.89 | 0.89 | 3.98 |  |  |
| VDD | IVW（fixed effects） | 0.686 | 1.84 | 0.09 | 35.98 | 0.050 | 0.902 |
|  | IVW（random effects） | 0.766 | 1.84 | 0.03 | 103.39 |  |  |
|  | Weighted median | 0.996 | 0.99 | 0.01 | 71.79 |  |  |
|  | MR Egger | 0.956 | 0.60 | 0.00 | 16.58 |  |  |
|  | Maximum likelihood | 0.682 | 1.88 | 0.09 | 39.21 |  |  |
| gout | IVW（fixed effects） | 0.005 | 2.74 | 1.35 | 5.59 | 0.840 | 0.612 |
|  | IVW（random effects） | 0.000 | 2.74 | 1.60 | 4.69 |  |  |
|  | Weighted median | 0.038 | 2.69 | 1.06 | 6.83 |  |  |
|  | MR Egger | 0.272 | 5.99 | 0.30 | 120.14 |  |  |
|  | Maximum likelihood | 0.006 | 2.77 | 1.35 | 5.71 |  |  |
| hyperthyroidism | IVW（fixed effects） | 0.774 | 1.10 | 0.58 | 2.06 | 0.647 | 0.178 |
|  | IVW（random effects） | 0.745 | 1.10 | 0.63 | 1.92 |  |  |
|  | Weighted median | 0.457 | 1.39 | 0.58 | 3.33 |  |  |
|  | MR Egger | 0.171 | 7.27 | 0.53 | 99.40 |  |  |
|  | Maximum likelihood | 0.769 | 1.10 | 0.58 | 2.08 |  |  |
| hypothyroidism | IVW（fixed effects） | 0.000 | 1.97 | 1.42 | 2.72 | 0.466 | 0.067 |
|  | IVW（random effects） | 0.000 | 1.97 | 1.43 | 2.71 |  |  |
|  | Weighted median | 0.002 | 2.01 | 1.29 | 3.14 |  |  |
|  | MR Egger | 0.038 | 86.58 | 2.55 | 36.02 |  |  |
|  | Maximum likelihood | 0.000 | 2.01 | 1.43 | 2.82 |  |  |
| HTN | IVW（fixed effects） | 0.000 | 2.17 | 1.68 | 2.81 | 0.224 | 0.347 |
|  | IVW（random effects） | 0.000 | 2.17 | 1.62 | 2.91 |  |  |
|  | Weighted median | 0.000 | 2.30 | 1.56 | 3.38 |  |  |
|  | MR Egger | 0.057 | 4.00 | 1.15 | 13.83 |  |  |
|  | Maximum likelihood | 0.000 | 2.23 | 1.70 | 2.93 |  |  |

Abbreviations: SNPs, single nucleotide polymorphisms; or, odds ratio; lci, lower confidence interval; uci, upper confidence interval; IVW, inverse-variance weighted; MR, Mendelian randomization; T2DM, type 2 diabetes mellitus; OS, osteoporosis; VDD, vitamin D deficiency; HTN, hypertension.

**Supplementary Figure 1 Core assumptions of Mendelian randomization.**


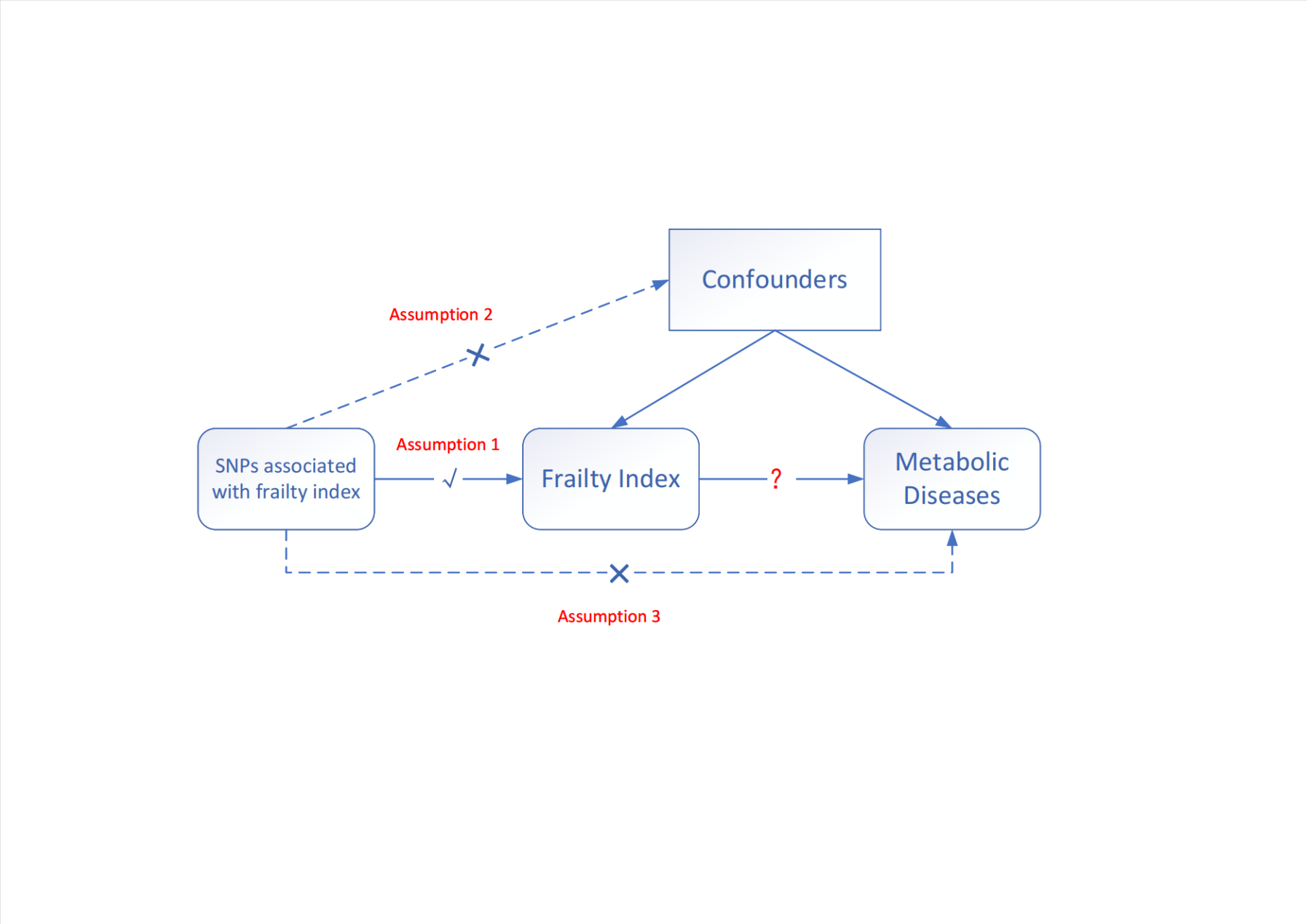


Abbreviation: SNPs, single-nucleotide polymorphisms.

**Supplementary Figure 2 Scatter plots of frailty index for obesity, type 2 diabetes, gout, hypothyroidism, and hypertension.**

**
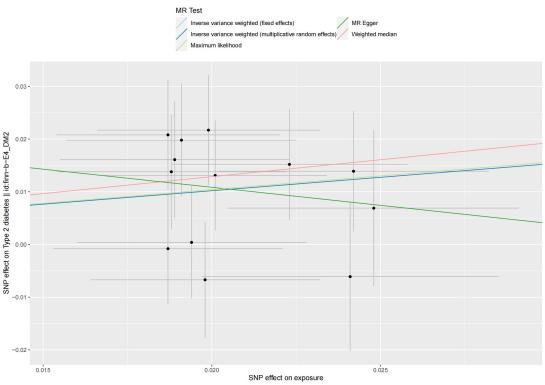

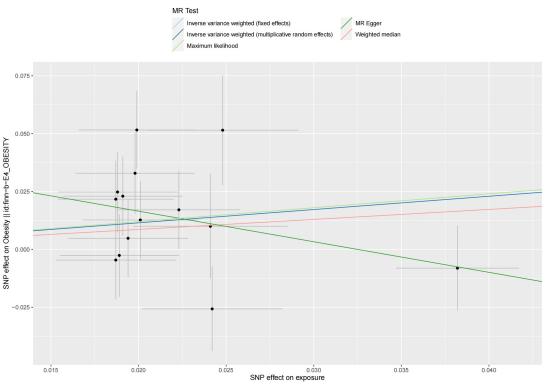

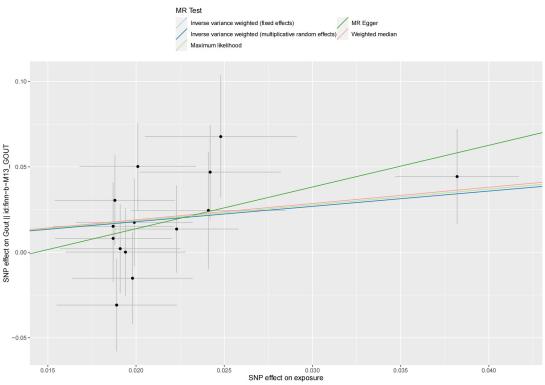
**

a b c

**
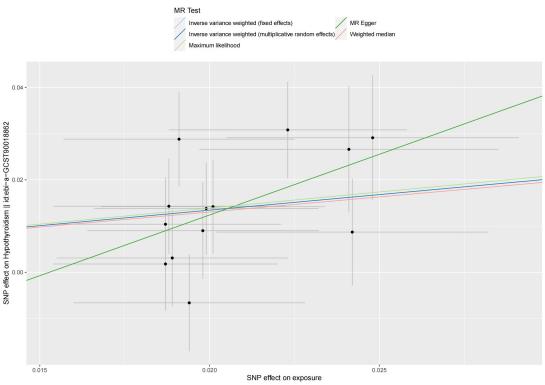

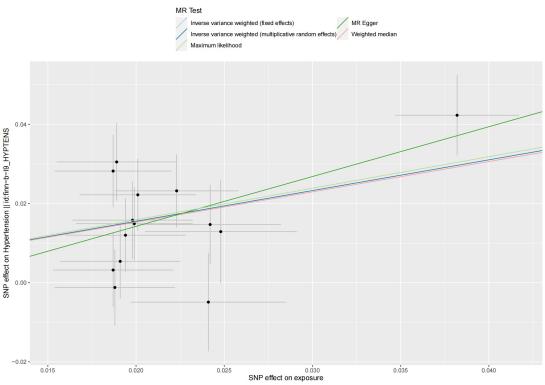
**

d e

Note: a, SNP effects on FI against the SNP effects on obesity; b, SNP effects on FI against the SNP effects on type 2 diabetes mellitus; c, SNP effects on FI against the SNP effects on gout; d, SNP effects on FI against the SNP effects on hypothyroidism; e, SNP effects on FI against the SNP effects on hypertension.

**Supplementary Figure 3 leave-one-out plots of frailty index for obesity, type 2 diabetes, gout, hypothyroidism, and hypertension.**

**
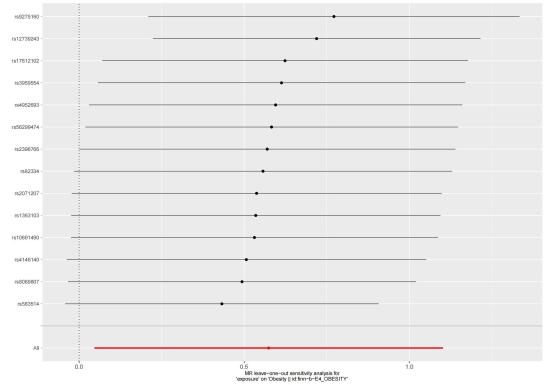
**

**
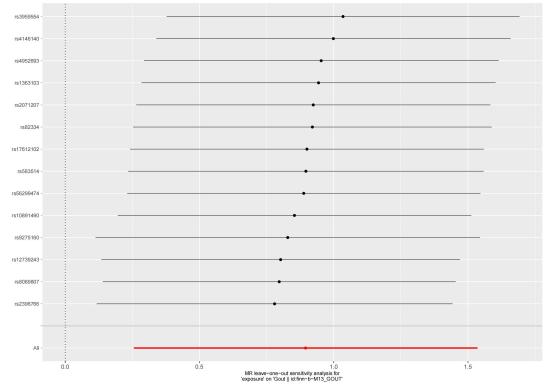

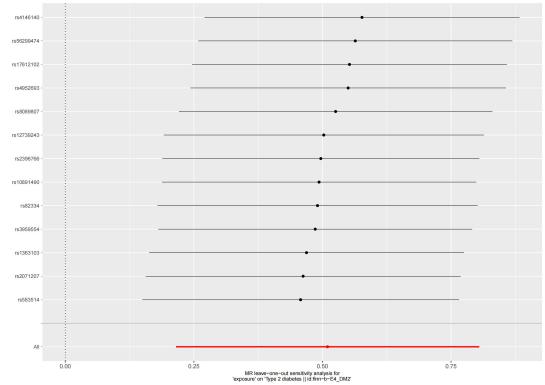
**

a b c

**
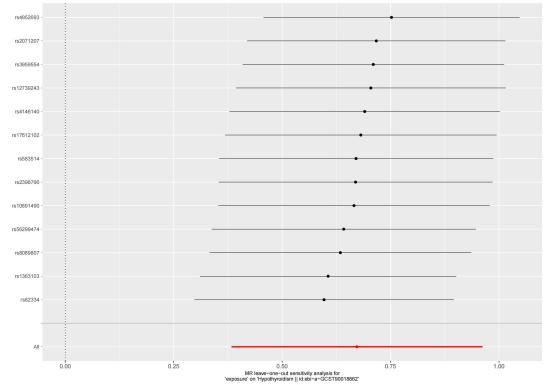

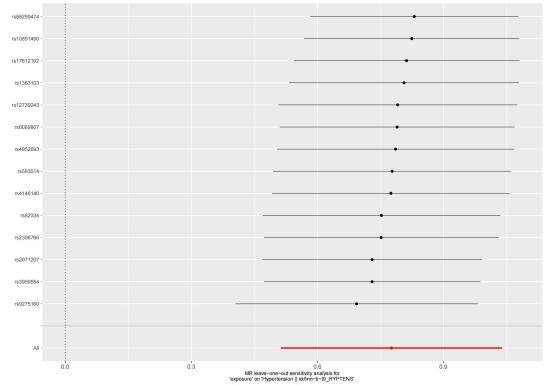
**

d e

Note: a, MR leave-one-out sensitivity analysis for FI on obesity; b, MR leave-one-out sensitivity analysis for FI on type 2 diabetes mellitus; c, MR leave-one-out sensitivity analysis for FI on gout; d, MR leave-one-out sensitivity analysis for FI on hypothyroidism; e, MR leave-one-out sensitivity analysis for FI on hypertension.
